# The Effects of Fennel Essential Oil Supplementation on Mitigating the Heat Stress Impacts on Growth Rate, Blood Biochemical Parameters, and Liver Histopathology in Broiler Chickens

**DOI:** 10.3390/vetsci12090825

**Published:** 2025-08-27

**Authors:** Shimaa A. Amer, Ahmed Gouda, Rehab I. Hamed, Arwa H. Nassar, Hanaa S. Ali, Rania M. Ibrahim, Gehan N. Alagmy, Azza M. M. Abdelmoteleb, Fayez Althobaiti, Khalid S. Alotaibi, Shatha B. Albattal, Mohamed Mohamed Soliman, Saed A. Althobaiti, Gehan K. Saleh

**Affiliations:** 1 Department of Nutrition and Clinical Nutrition, Faculty of Veterinary Medicine, Zagazig University, Zagazig 44511, Egypt; 2 Animal Production Department, Agricultural & Biological Research Division, National Research Center, Dokki, Cairo 11865, Egypt; 3 Department of Poultry Diseases, Reference Laboratory for Quality Control on Poultry Production (RLQP), Animal Health Research Institute (AHRI), Zagazig Branch, Agriculture Research Center (ARC), Dokki, P.O. Box 246, Giza 12618, Egypt; 4 Food Hygiene Department, Animal Health Research Institute (AHRI) (Mansoura Branch), Agriculture Research Center (ARC), Dokki, P.O. Box 246, Giza 12618, Egypt; 5 Department of Pathology, Animal Health Research Institute (AHRI) (Mansoura Branch), Agriculture Research Center (ARC), Dokki, P.O. Box 246, Giza 12618, Egypt; 6 Department of Pathology, Animal Health Research Institute (AHRI) (Zagazig Branch), Agriculture Research Center (ARC), Dokki, P.O. Box 246, Giza 12618, Egypt; 7 Department of Biochemistry, Toxicology, and Feed Deficiency, Pharmacology and Pyrogen Unit, Animal Health Research Institute (AHRI), Agriculture Research Center (ARC), Dokki, P.O. Box 246, Giza 12618, Egypt; 8 Department of Biotechnology, College of Science, Taif University, P.O. Box 11099, Taif 21944, Saudi Arabia; 9 General Science and English Language Department, College of Applied Sciences, AlMaarefa University, Riyadh 11597, Saudi Arabia; 10 Clinical Laboratory Sciences Department, Turabah University College, Taif University, P.O. Box 11099, Taif 21944, Saudi Arabia; 11 Department of Biology, Turabah University College, Taif University, Turabah, P.O. Box 11099, Taif 21944, Saudi Arabia; 12 Department of Biochemistry, Animal Health Research (AHRI) (Mansoura Branch), Agriculture Research Center (ARC), Dokki, P.O. Box 246, Giza 12618, Egypt

**Keywords:** antioxidants, broiler chickens, growth, heat stress, liver histology

## Abstract

The current study evaluated the role of dietary fennel essential oil (FO) supplementation in ameliorating the effects of heat stress on growth performance, meat quality, antioxidant activity, inflammatory responses, and liver histopathology in broiler chickens. Six hundred male broiler chicks (three-day-old ROSS broilers) were allocated into six experimental treatments (TRTs); the first was the negative control (Neg. CON, not subjected to hot temperature conditions), and the second was the positive control group (PS CON, subjected to hot temperatures). The control groups (Neg. and PS) were fed the basal diet without supplements. The third, fourth, and fifth groups were fed diets supplemented with 1 g FO/kg diet, 2 g FO/kg diet, and 3 g FO/kg diet, respectively and subjected to hot temperatures. The sixth group was fed the basal diet, received 500 mg of paracetamol per liter of drinking water, and subjected to hot temperatures. Hot temperature conditions (36 ± 0.5 °C) was applied for 6 h/day from the 22nd to the 25th day of the feeding period. The feeding period lasted for 35 days. The results showed that FO supplementation improved the body weight, weight gain, and feed conversion ratio compared to those in the PS CON and paracetamol groups. The growth hormone concentrations increased in the FO-supplemented TRTs and the paracetamol groups compared to those in the Neg. and PS CON groups. The serum total protein, albumin, and globulin concentrations significantly increased in the FO-supplemented TRTs compared to those in the Neg. and PS CON groups and the paracetamol TRTs. The serum TAC increased in the 3 g FO/kg TRT. The serum activity of CAT and SOD increased in the 3 and 2 g FO/kg TRTs and the paracetamol TRTs compared to those in the Neg. and PS CON groups. The serum MDA concentrations decreased in the FO-supplemented TRTs and paracetamol groups compared to those in the Neg. and PS CON groups. The IL1β and IFN-α concentrations decreased in the FO-supplemented and paracetamol groups compared to those in the PS CON groups. The HSP70 concentration was the highest in the 3 g FO/kg TRT. The immune expression of IL1-β and TGF-β in the liver tissues was downregulated in the FO-supplemented groups, especially the FO3 group, compared to those in the PS CON group. In conclusion, dietary supplementation with FO increased the broiler chickens’ growth more than that in the PS CON and paracetamol groups under hot temperatures. Fennel oil supplementation (3 g/kg diet) can alleviate the negative impacts of heat stress on broiler chickens’ antioxidant and inflammatory responses.

## Simple Summary

Heat stress has harmful impacts on poultry health, welfare, and production. So, different approaches have been used to mitigate these effects. The current study assessed the role of dietary fennel essential oil (FO) supplementation at three levels—1, 2, or 3 g/kg diet—compared with that of the traditional medication (paracetamol). This study’s outcomes showed that FO supplementation at the level of 3 g/kg diet can alleviate the negative impacts of heat stress on broiler chickens’ growth performance, antioxidant, and inflammatory responses. The supplementation with FO improved the broiler chickens’ growth performance and immunity more than those in the positive control and paracetamol groups during hot temperatures.

## 1. Introduction

Ecological temperatures are a key factor that influences poultry production. A comfort region of 16–25 °C exists for poultry species under normal circumstances [1]. When an animal is exposed to temperatures beyond this zone, it suffers from heat stress and cannot regulate its body temperature due to a lack of feathering or sweat glands [2,3,4]. According to the duration of exposure, heat stress is categorized into three types: acute, cyclic chronic, and constant chronic [2]. Heat stress has harmful impacts on poultry health, welfare, and production; nevertheless, the duration of stress matters [5,6,7,8,9]. Conversely, chronic heat stress often causes reactions dissimilar to acute reactions. Homeostatic controllers of the nervous and endocrine systems regulate acute heat stress, which lasts from a few hours to a few days. In contrast, homeostatic monitors of the endocrine system show a fundamental function in chronic heat stress, which lasts from several days to weeks [6,10].

Specific physiological alterations arise in birds when they are subjected to heat stress. Reactive oxygen and nitrogen species (ROS/RNS) are produced during heat stress and are responsible for catalyzing numerous reactions. Oxidative/nitrosative metabolism is significant for cell persistence [11,12]. ROS/RNS are naturally produced in all cells during cellular processes. Enzymatic and non-enzymatic antioxidant mechanisms cooperate to eliminate them from the cells if they accumulate excessively. This process is precisely manipulated by keeping a stable balance between oxidants and antioxidants. Nonetheless, when ROS/RNS are excessively produced, they interfere with the antioxidant capacity of the cells, causing numerous harmful effects, such as DNA degradation, lipid peroxidation, and protein carbonylation [13].

Feed additives added to poultry diets have been broadly used to alleviate the influences of heat stress [14,15]. Although various sources have been used to lessen the effects of heat stress, the use of herbal essential oils has become a debated issue in recent periods [16,17,18,19]. Essential oils consist of lipophilic, highly volatile secondary metabolites extracted through hydrodistillation. They include diverse compounds like monoterpenes, sesquiterpenes, and diterpenes. Essential oils represent an environmentally friendly food, medicine, and agriculture alternative due to their proven antimicrobial, antiviral, antinematode, antifungal, insecticidal, and antioxidant properties [20,21,22,23,24].

Fennel (*Foeniculum vulgare* Mill.), belonging to the Apiaceae (Umbelliferaceae) family, is an edible, aromatic plant with yellow flowers and pinnate leaves [25]. For many years, fennel has been used as a herbal drug in traditional and alternative medicine [26]. It has been reported that fennel feeding increases the weight and enhances the feed efficiency of broiler chickens [27]. Its essential oil can be extracted and is called fennel oil (FO) [28]. Fennel oil is a rich source of phenolics; flavonoids; anethole; camphene; phellandrene; fenchone; limonene; anisic acid; pinene; methyl chavicol; and oleic, linoleic, palmitic, and petroselenic acids [27,29,30]. Fennel oil has antimicrobial, anti-inflammatory [31], antioxidant [32,33], and hepatoprotective activities [34].

However, there are insufficient reports on using FO to alleviate heat stress compared to synthetic treatments such as paracetamol. To fill this research gap, this study was designed to understand the effect of FO oil as a stress reliever compared to that of the conventional treatment (paracetamol) on the growth performance, meat quality, antioxidant activity, inflammatory responses, and tissue histology of heat-stressed broiler chickens, given that these parameters are related to chicken health and productivity, especially under periods of heat stress.

## 2. Materials and Methods

### 2.1. Gas Chromatography–Mass Spectrometry (GC-MS) Analysis of FO

Fennel oil was obtained from Imtenan for Natural Products (Nasr City, Cairo, Egypt). The active compounds in FO were determined using a Trace GC-1310-ISQ Mass Spectrometer (Thermo Scientific, Austin, TX, USA), with a TG–5MS direct capillary column (30 m × 0.25 mm × 0.25 µm film thickness), as described by Amer et al. [23].

**Figure 1:**
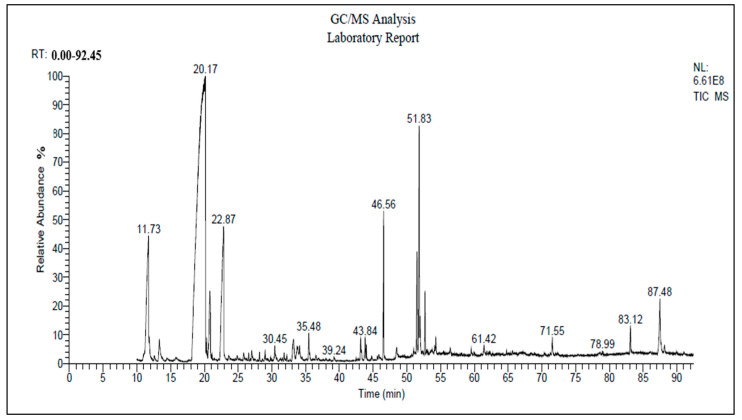
Chromatographic characteristics of FO compounds.

The main bioactive compounds include anethole (17.44%), estragole (16.57%), D-Limonene (13.41%), 9-Octadecenoic acid (Z)-, methyl ester (8.36%), Hexadecenoic acid, and methyl ester (5.19%) (Table 1 and Figure 1).

### 2.2. The Birds, Experimental Design, and Diets

This research was carried out in a poultry research unit in the faculty of veterinary medicine, Zagazig University, Egypt, to assess the role of dietary FO supplementation in ameliorating the effects of heat stress on the growth performance, antioxidant activity, inflammatory responses, and intestinal histomorphology of broiler chickens. All of the experimental procedures were approved by the ARC-IACUC committee (Approval No. ARC-IACUC/AHRI/142/24).

Six hundred one-day-old male broiler chicks were obtained from a commercial chick producer. Before the start of the experiment, the birds underwent a three-day acclimatization period to reach a mean weight of 103.39 g ± 3.37 g. Broiler chicks were randomly assigned into 6 experimental groups for the 35-day feeding period; the first was the negative control (Neg. CON, not subjected to hot temperature conditions), and the second was the positive control group (PS CON, subjected to hot temperature conditions). The control groups (Neg. and POS) were fed the basal diet without supplements. The 3rd, 4th, and 5th groups were fed diets supplemented with 1 g of FO/kg diet, 2 g of FO/kg diet, and 3 g of FO/kg diet and subjected to hot temperatures. The 6th group was fed the basal diet, received 500 mg paracetamol per liter of drinking water, and subjected to hot temperatures. The normal brooding temperature was adjusted to 34 °C and was gradually decreased until it reached 25 °C at the end of the rearing period, as recommended for ROSS broiler chickens [35]. The groups exposed to acute heat stress were placed under an environmental temperature of 36 ± 0.5 °C for 6 h/day from the 22nd to the 25th day of the feeding period. The temperature was maintained within this range using a heater–air conditioning system. The relative humidity ranged from 68.5 to 70.5% during the heat stress periods. Fennel oil was mixed with feed ingredients and fed to the birds in mash form. The feeding period was divided into the following three periods: starter (the 4th–10th day), grower (the 11th–23rd day), and finisher (the 24th–35th day). Throughout the experiment, feed and water were added ad libitum. The ration formulations for each feeding period (starter, grower, and finisher) (Table 2) and the rearing conditions were adjusted according to AVIAGEN [35].

### 2.3. The Growth Performance

The broiler chickens were weighed individually on day 4 of age to obtain their average initial body weight, and their body weight was then recorded on days 10, 23, and 35 to calculate the average body weight of the birds in each group.

The body weight gain (BWG) was calculated as follows:

BWG=W2−W1

W2 is the final body weight in the intended period; and W1 is the initial body weight in the same period.

The feed intake (FI) for each replicate was documented as the difference between the weight of feed provided and the remaining residues. Then, the average feed intake per bird was divided by the number of birds in each replicate to find the average feed intake per bird.

The feed conversion ratio (FCR) was calculated as follows:

$$FCR=\frac{FI\;(g)}{BWG\;(g)}$$

### 2.4. The Meat Quality and Chemical Composition of the Breast Muscle

At the end of the experiment, ten broiler chickens from each group were euthanized through cervical dislocation [36]. A trained 5-person descriptor panel determined the sensory attributes (color, aroma, and texture) of the examined muscles and assigned a score from 1 to 5, where 5 represented normal, 4 represented slight deviation, 3 represented moderate deviation, 2 represented major deviation, and 1 represented severe deviation. The broiler chickens’ muscles were sampled from poultry carcasses to determine the pH, thawing losses, and cooking (samples were kept in a preheated water bath for 10 min to reach 75 °C), according to Petracci and Baéza [37].

The chemical composition of the breast muscles (5 samples/group), including dry matter, fat, crude protein, and ash content %, was determined according to AOAC [38].

### 2.5. Sample Collection and Laboratory Analyses

On the 35th day, blood samples were randomly collected after slaughter (ten chickens/group). The chicks were euthanized using cervical dislocation, according to the American Veterinary Medical Association guidelines [36]. Blood samples were collected without anticoagulants, allowed to clot at room temperature, centrifuged for 15 min at 3500 rpm to separate the serum, and stored at −20 °C in a deep freezer until the biochemical analysis. Liver samples (10 samples/group) were taken and kept at −20 °C for the heat shock protein 70 (HSP70) analysis. The other liver samples were taken for histomorphology and immunohistochemistry studies.

### 2.6. Blood Biochemical Indices

Growth hormone (GH) was determined using chicken ELISA kits from My BioSource Co., San Diego, CA, USA, with Cat. No. MBS266317. The glucose, creatinine, and uric acid serum levels were measured using an automatic biochemical analyzer (Robonik Prietest ECO, Navi Mumbai, India) [39,40,41]. Reitman and Frankel [42] was used to estimate serum levels of aspartate aminotransferase (AST) and alanine aminotransferase (ALT). The serum levels of total protein and albumin were determined according to Grant [43] and Doumas, et al. [44], respectively. Serum globulin levels were calculated mathematically by subtracting the albumin value from the total protein value [45].

### 2.7. Antioxidant Activity and Inflammatory Responses

We estimated the serum total antioxidant capacity (TAC) [46]; catalase (CAT) [47] and superoxide dismutase (SOD) activity [48]; and malondialdehyde (MDA) levels [49]. Interferon γ (INF-γ) and interleukin 1β (IL-1β) levels were measured using ELISA assay kits (MyBioSource, San Diego, CA, USA) (Cat. Nos. MBS700243 and MBS2024496, respectively). An enzyme-linked immunoassay for HSP70 in the liver tissues was used [50].

### 2.8. Histopathological Examination of the Liver

Liver specimens (10 samples/group) were collected from the chickens and fixed in 10% neutral-buffered formalin for analysis. The tissue samples were dehydrated in gradually increasing ethanol concentrations (75–100%). Then, they were submerged in xylol I and II before being embedded in paraffin. Cross-sectional and longitudinal sections were cut into 4 µm using a microtome (Leica RM 2155 Nussloch, Germany,). These sections were stained using hematoxylin and eosin (H&E) [51]. The sections were photographed under high-power magnification (×200) using an AmScope 5.0 MP microscope digital camera (25 images for each group).

### 2.9. The Immunohistochemical Procedures

The liver sections (ten samples/group) were examined for inflammatory mediators, specifically IL-1β and TGF-β [52]. Endogenous peroxidase blocking reagent containing hydrogen peroxide and sodium azide was applied to the tissue sections (DAKO peroxidase blocking reagent, Cat. No. S 2001). Next, one to two drops of the supersensitive primary monoclonal antibody against IL-1β (Cat. NBP3-11364) and TGF-β (Cat. BAF240) (Novus Biologicals, Briarwood Avenue, Centennial, CO, USA) were added to these sections; the slides were then stained with hematoxylin and observed under a microscope. The morphometric analysis was performed using Image J software (version 1.45) (bundled with 64-bit Java 1.8.0_172, National Institutes of Health, Bethesda, MD, USA) to accurately measure various immune-positive cells and their proportions in the livers from the tested chicken groups in three high-power fields [53].

### 2.10. Statistical Analysis

The data were analyzed with a one-way analysis of variance (ANOVA) using the GLM procedure in SPSS Version 16 for Windows (SPSS Inc., Chicago, IL, USA) after Shapiro–Wilk’s test was used to verify the normality and Levene’s test was used to verify the homogeneity of variance components between experimental treatments. Tukey’s test compared the differences between the means at 5% probability. The data variance was expressed as the pooled SEM, and the significance level was set at *p* < 0.05.

## 3. Results

### 3.1. Growth Performance

During the starter period, the experimental groups did not significantly differ in the growth performance parameters (*p* > 0.05). During the grower period, there was a significant decrease in the BW in the 2 and 3 g FO/kg TRTs and the paracetamol TRT compared to that in the Neg. CON group (*p* < 0.01), while the BWG was decreased in the 3 g FO/kg and paracetamol TRTs compared to that in the other groups (*p* < 0.05). The feed intake was reduced in the FO-supplemented TRTs (*p* < 0.01). The FCR was increased in the 3 g FO/kg and paracetamol TRTs compared to that in the Neg. CON group (*p* < 0.01). During the finisher period, the BW, BWG, and FCR were improved in the FO-supplemented groups compared to these values in the PS CON group (*p* < 0.01). The FCR was increased in the PS CON and paracetamol TRTs compared to that in the Neg. CON group (*p* < 0.01). The overall performance showed that FO supplementation improved the BW, BWG, and FCR compared to those in the PS CON group during the heat stress period (*p* < 0.01) (Table 3).

### 3.2. The Physical Characteristics of the Meat

**Figure 2:**
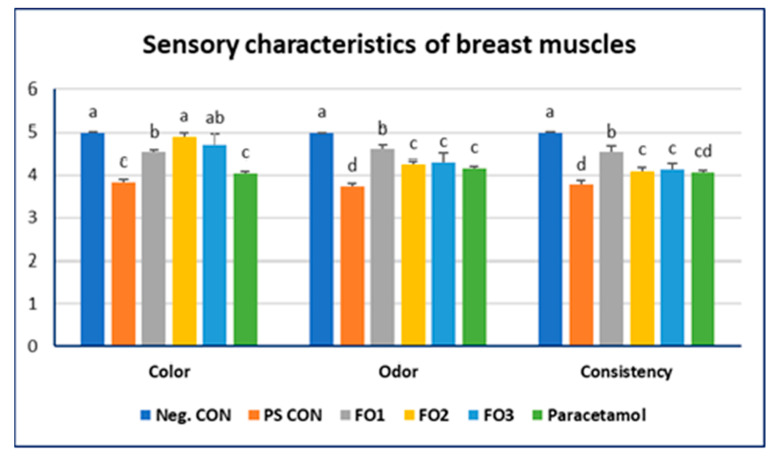
The effect of fennel oil on the sensory characteristics of broiler carcasses. Bars with different letters (a–d) indicate significant differences (*p* < 0.05).

Sensory characteristics, including odor, color, and consistency (Figure 2), showed a significant difference between the experimental groups, where FO2 and FO3 showed the same color as that in the Neg. CON group (*p* < 0.01). The FO-supplemented groups showed better color, odor, and consistency than those in PS CON (*p* < 0.01) (Figure 2). The shear value, used in the texture evaluation, decreased in the fennel-oil- and paracetamol-supplemented groups compared to that in the negative and positive control groups (*p* < 0.01). The PH value did not significantly differ between the experimental groups (*p* > 0.05). Increased cooking loss and decreased thawing loss were detected in the PS CON group compared with those in the Neg. CON group (*p* < 0.01). Lightness was higher in the FO3 and paracetamol groups compared with that in the other experimental groups (*p* < 0.01). Redness was not significantly different between groups (*p* > 0.05). Yellowness decreased in all experimental groups except the FO2 group compared with that in the Neg. CON group (*p* < 0.01) (Table 4).

### 3.3. The Chemical Composition of the Breast Muscle

The moisture content of the breast muscle was increased in the paracetamol TRT, followed by the values in 3 g FO/kg, 1 g FO/kg, 2 g FO/kg, PS CON, and Neg. CON (*p* < 0.01). The crude protein content was increased in the 1 g FO/kg TRT compared to that in the other groups (*p* > 0.05). The ash content was increased in the PS CON, 1 g FO/kg, 3 g FO/kg, and paracetamol TRTs compared to that in Neg. CON (*p* < 0.01)) (Table 5).

### 3.4. Serum Biochemical Parameters

The growth hormone concentrations increased in descending order as follows: those in the 3 g FO/kg, 2 g FO/kg, 1 g FO/kg, and paracetamol TRTs compared to those in the Neg. and PS CON groups (*p* < 0.01). The serum total protein, albumin, and globulin concentrations significantly increased in the 3 g FO/kg, 2 g FO/kg, and 1 g FO/kg TRTs compared to those in the Neg. and PS CON groups and the paracetamol TRT (*p* < 0.01). The albumin/globulin ratio decreased significantly in the 3 g FO/kg and 2 g FO/kg TRTs compared to that in the PS CON group (*p* < 0.01). The concentrations of glucose, AST, ALT, creatinine, and uric acid were not significantly different between the experimental TRTs (*p* > 0.05) (Table 6).

### 3.5. Antioxidant Capacity and Inflammatory Indices

The serum TAC increased in the 3 g FO/kg TRT compared to that in the other experimental TRTs, followed by the 2 g FO/kg, 1 g FO/kg, and paracetamol TRTs (*p* < 0.01). The serum activity of CAT and SOD increased in the 3 g FO/kg, 2 g FO/kg, and paracetamol TRTs compared to that in the Neg. and PS CON groups (*p* < 0.01). The serum MDA concentrations decreased in the FO-supplemented TRTs and the paracetamol TRTs compared to those in the Neg. and PS CON groups (*p* < 0.01). The HSP70 concentrations were the highest in the 3 g FO/kg TRT compared to those in the other experimental groups (*p* < 0.01). The IL1β and IFN-α concentrations decreased in the FO-supplemented groups and paracetamol groups compared to those in the PS CON group (*p* < 0.01) (Table 7).

### 3.6. The Histopathological and Immunohistochemical Findings

**Figure 3:**
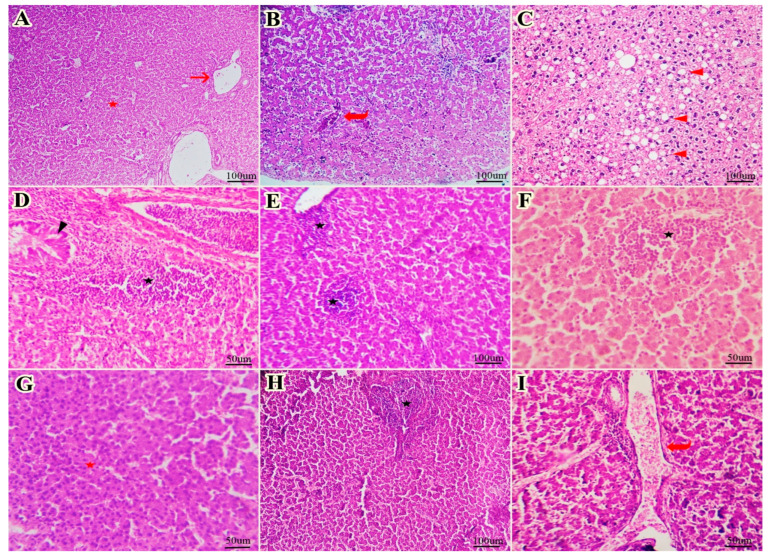
Histomorphological examination of the livers in the experimental groups. (**A**) The Neg. CON group showed healthy hepatic cellular architecture (red star) and vascular tissues (arrow). (**B**–**D**) The PS CON group showed dilated hepatic vasculature (curved arrow), fatty degenerations within numerous hepatocytes (arrowheads), and focal leucocytic aggregations within the portal areas (black star) accompanied by hyperplasia of the bile duct epithelium (black arrowhead). (**E**) The FO1 group showed multiple foci of inflammatory cell infiltrates (black star). (**F**) The FO2 group showed aggregates of inflammatory cells primarily in the perivascular tissues (black star). (**G**) The FO3 group showed preserved hepatic cellular architecture (red star) and vascular tissue. (**H**,**I**) The paracetamol group showed inflammatory cell infiltrates primarily in the portal areas (black star) beside dilated hepatic blood vessels (curved arrow) (**H**,**E**).

The examined liver sections from the Neg. CON group showed healthy hepatic cellular architecture and vascular tissues (Figure 3A). The examined liver sections from the PS CON group showed dilated hepatic vasculature, fatty degenerations within numerous hepatocytes, and focal leucocytic aggregations within the portal areas, accompanied by hyperplasia of the bile duct epithelium (Figure 3B–D). The examined liver sections from the FO1 group showed multiple foci of inflammatory cell infiltrates (Figure 3E). The examined liver sections from the FO2 group showed aggregates of inflammatory cells, primarily in the perivascular tissues (Figure 3F). The examined liver sections from the FO3 group showed preserved hepatic cellular architecture and vascular tissues (Figure 3G). The examined liver sections from the paracetamol group showed inflammatory cell infiltrates primarily in the portal areas along with dilated hepatic blood vessels (Figure 3H,I).

**Figure 4:**
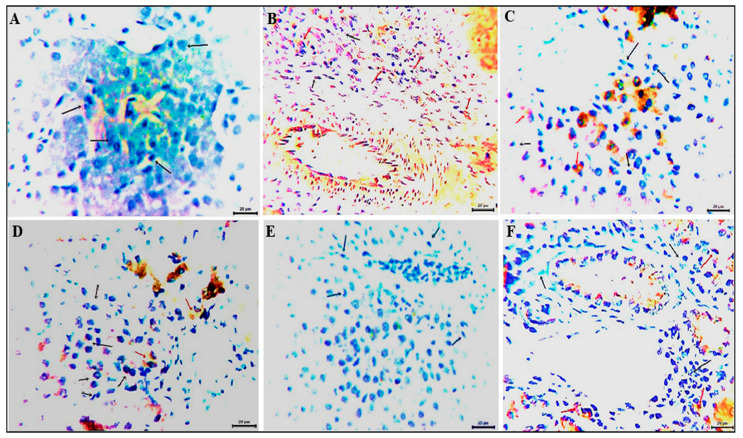
Photomicrographs from chicken livers immune-stained with a monoclonal antibody against the pro-inflammatory cytokine IL1-β showing the percentage of the expressed antigen as a brown cytoplasmic staining reaction of moderate intensity (red arrows). Black arrows point to negative cells. (**A**) Neg. CON group; (**B**) PS CON group; (**C**) FO1 group; (**D**) FO2 group; (**E**) FO3 group; (**F**) paracetamol group. Scale bar: 20 μm.

**Figure 5:**
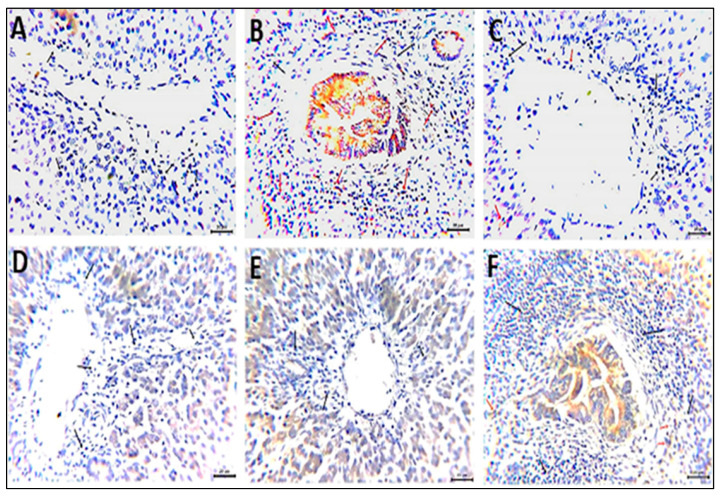
Photomicrographs from chicken livers immunostained using a monoclonal antibody against the TGF-β surface receptor antigen showing the percentage of the expressed antigen as a brown cytoplasmic staining reaction of moderate intensity (red arrows). Black arrows point to negative cells. (**A**) Neg. CON group; (**B**) PS CON group; (**C**) FO1 group; (**D**) FO2 group; (**E**) FO3 group; (**F**) paracetamol group. Scale bar: 20 μm.

Sections from the chickens’ liver tissues immune-stained against the pro-inflammatory cytokine IL1-β revealed expression levels of zero, 5–8, 1.5–2.6, 0.5–1, zero, and 0.5–1% in the Neg. CON, PS CON, FO1, FO2, FO3, and paracetamol groups, respectively (Figure 4). Examined sections from the chicken liver tissues immune-stained with specific monoclonal antibodies against the TGF-β surface receptor antigen demonstrated cytoplasmic expression of zero, 38–46, 0.5, zero, zero, and 0.5% in the corresponding groups, with moderate to intense staining reactivity (Figure 5).

## 4. Discussion

Adaptation to heat stress significantly affects broiler performance and the economic efficiency production index [14,54]. Although some animal species are sensitive to heat stress, poultry, especially novel breeds, are more sensitive to high environmental temperatures, which has significant consequences for the poultry industry, as heat stress produces substantial economic losses [18]. Heat stress negatively impacts various attributes of poultry, including their physiological responses and productive and reproductive performance. These impacts occur in specific molecular and metabolic routes. To lessen the effects of heat stress, it is important to go beyond management practices and implement nutritional interventions during elevated ambient temperatures. In the current study, we employed an acute heat stress model to evaluate the role of dietary fennel essential oil (FO) supplementation in alleviating heat stress’s effects on the growth performance, meat quality, antioxidant activity, and inflammatory responses in broiler chickens in comparison with those under conventional treatment (paracetamol). As reported in our study, the main bioactive compounds in FO are anethole (17.44%), estragole (16.57%), D-Limonene (13.41%), 9-Octadecenoic acid (Z)-, methyl ester (8.36%), Hexadecenoic acid, and methyl ester (5.19%).

Heat stress influences feed consumption, behavior, and nutrient digestion via different mechanisms [18]. It may result in a physiological imbalance that stimulates the body to use nutrients for protein synthesis rather than growth, giving broiler chickens less resistance to the oxidative damage caused by heat stress [54,55,56]. A decreased feed intake is one of these animals’ first responses to heat stress. A decreased feed intake negatively affects parameters such as BW, BWG, digestive enzyme secretion, and nutrient absorption, eventually compromising the feed conversion ratio. These unfavorable changes also disturb other production factors in poultry [16,57]. This study revealed that FO supplementation (1–3 g/kg diet) improved the broiler chickens’ BW, BWG, and FCR compared to those in the PS CON and paracetamol groups during hot temperatures. It has been shown that heat stress causes a decrease in the relative weight of the carcass and digestive, reproductive, and immune organs [3,58]. One of the most important factors contributing to the affirmative impacts of essential oils on growth and productivity is their ability to promote digestion. Essential oils are often reported to improve the flavor and taste of feed [59]. Essential oils stimulate the feed intake response and boost secretory activity (e.g., saliva, bile acids, gastric, and pancreatic enzymes) in the digestive tract by initiating sensory centers in the digestive tract through olfactory stimulation or the existence of specific bioactive compounds [59,60]. These factors also justify the increased crude protein content in the breast muscles of the broiler chickens that received 1 g FO/kg compared to that in the other groups, which indicates improved protein utilization due to FO supplementation. In addition, the ash content was increased in the PS CON, 1 g FO/kg, 3 g FO/kg, and paracetamol TRTs compared to that in Neg. CON. Fennel oil plays an important role in enhancing digestion by encouraging the secretion of digestive fluids, stimulating enzymes, and inhibiting the effects of pathogenic bacteria [32]. The improved growth performance in the current study may also be due to the increased growth hormone concentration in the FO-supplemented TRTs and the paracetamol group compared to those in the Neg. and PS CON groups. Al-Sagan, et al. [61] exhibited an increased growth rate in broiler chickens during chronic heat stress and improved redness in the breast meat due to dietary fennel seed powder supplementation during 19–41 days of age. Schöne, et al. [62] pointed out that the primary compound in fennel oil is anethole, representing 50–70%. It has been indicated that anethol promotes the growth performance [63] by activating the enzymes responsible for digestion [64].

Heat stress induces oxidative stress, which can significantly affect chicken meat quality. The elevated generation of reactive oxygen species (ROS) can lead to muscle aging, protein degradation, and the impairment of nuclear proteins, including DNA and RNA. The mitochondrial dysfunction caused by oxidative stress results in elevated ROS production, an impaired aerobic fat and glucose metabolism, and increased glycolysis [65]. Hence, adenosine triphosphate (ATP) production decreases; the calcium balance is disrupted; and proteins and lipids in the mitochondria are oxidized, leading to mitochondrial membrane disruption in the muscle cells. A malfunctioning aerobic metabolism leads to anaerobic glycolysis, causing the accumulation of H+ ions and lactic acid in the muscles, ultimately decreasing the pH [66,67]. pH level is a crucial factor impacting meat’s color attributes and water-holding capacity. A low pH in meat is associated with a pale color and increased drip loss [68]. These criteria encompass the assessment of color intensity through the utilization of lightness (L*) (L* = 0 for black, L* = 100 for white), redness (a*) (a* = +60 for red, a* = −60 for green), and yellowness (b*) (b* = +60 for yellow, b* = −60 for blue) values, within the CIELAB standards [69].

Essential oils have been demonstrated to improve color and textural properties by influencing the pH value of meat. Studies have indicated that dietary supplementation with essential oils increases the pH value while decreasing the L*, a*, and b* values in heat-stressed poultry, which indicates alterations in color intensities. Essential oils have shown these effects through preventing the breakdown and oxidation of lipids and proteins in meat, causing an improvement in fatty acid composition [16,70]. They help prevent the oxidation of monounsaturated fatty acids (ΣMUFA) and polyunsaturated fatty acids (ΣPUFA), which are susceptible to oxidation [71]. Furthermore, essential oils reduce the accumulation of lactic acid in the muscles and regulate the electrolyte balance in the blood [58]. These mechanisms improve the quality and oxidative stability of meat and thus enhance meat quality in heat-stressed poultry species.

The current study showed that the FO2 and FO3 groups showed the same color as that in Neg. CON. The FO-supplemented groups showed better color, odor, and consistency than these properties in PS CON. The pH values did not significantly differ between the experimental groups. Increased cooking loss and decreased thawing loss were detected in the PS CON group. The lightness of the muscles was higher in the FO3 and paracetamol groups. Redness was not significantly different between groups. Yellowness decreased in all experimental groups except the FO2 group. Meat’s color is related to the concentrations and condition of myoglobin and hemoglobin [72]. It is worth noticing that the a* value is important to consumers. Moreover, a high redness (a*) value gives an undercooked appearance. The a* value can be influenced by the bird’s age, pre-slaughter stress, and dietary nitrate intake [73]. Imbabi et al. [32] showed no difference in meat pH between different temperature and/or fennel content groups.

Low total protein, albumin, and globulin levels may suggest decreased immune activity under stressful conditions. Still, if their concentrations rise under stress-free conditions, this may imply that the proteins taken are being used for growth [74]. The current study showed that the serum total protein, albumin, and globulin concentrations significantly increased in the FO-supplemented TRTs compared to those in the Neg. and PS CON groups and the paracetamol TRT. It has been reported that supplementation with essential oils in the diet or drinking water of heat-stressed poultry species causes an increase in total protein, albumin, and globulin levels. Furthermore, the results showed that the concentrations of glucose, AST, ALT, creatinine, and uric acid were not significantly different between the experimental TRTs. These results indicate that acute heat stress did not negatively affect liver or kidney function. ALT and AST have been used as indicators of liver health [75]. Kumar and Nazir et al. [76,77] reported that *Funiculus vulgare* reduces ALT, alkaline phosphatase (ALP), and AST levels in the serum. These beneficial effects may be due to the ability of essential oils to increase antioxidant enzyme production, enhance organ and tissue function, and decrease protein degradation [70,78].

The activity of antioxidant enzymes and the concentrations of oxidative products are crucial for assessing oxidative status in poultry. Although several studies over the years have shown that heat stress encourages oxidative stress, the mechanisms through which essential oils improve antioxidant activity or reduce oxidative stress have only been examined recently [58,79]. Essential oils have direct and indirect antioxidant effects. Essential oils directly mitigate oxidative stress through high reactivity with peroxyl radicals and are removed by transferring formal hydrogen atoms. Essential oils contain phenolic hydroxyl groups, which inhibit the formation of hydroperoxide from the peroxyl radicals produced in the early stage of lipid oxidation [70]. Essential oils employ their effects indirectly through several mechanisms, including regenerating antioxidant enzymes; enhancing the activity of antioxidant enzymes; and modulating other defense pathways, such as the activation of heat shock proteins, detoxification, and apoptosis processes [70,79,80]. Heat shock proteins (HSPs), made by all organisms due to excessive heat, act as chaperones during stressful conditions to maintain cell integrity by detecting denatured or damaged proteins and directing them toward degradation [81]. MDA concentration is one of the most valuable biomarkers for lipid peroxidation. SOD and CAT play an important role in scavenging free radicals from the cells [82]. The current study showed that the serum TAC increased in the 3 g FO/kg TRT; the serum activity of CAT and SOD increased in the 3 and 2 g FO/kg TRTs and the paracetamol TRT; and the serum MDA concentrations decreased in the FO-supplemented TRTs and the paracetamol TRT. The IL1β and IFN-α concentrations decreased in the FO-supplemented and paracetamol groups compared to those in the PS CON group. The HSP70 concentrations were the highest in the 3 g FO/kg TRT.

Furthermore, our study assessed the extent of tissue injury in the heat stress group by examining liver histomorphology. Inflammatory infiltration was observed in the heat-stressed groups. The results showed that heat stress induced liver tissue damage. Earlier studies have reported that heat stress is responsible for injury and oxidative stress in chicken tissue [83]. While inflammation is an important indicator of tissue injury or damage in the respective organs, findings have depicted several alterations in the normal histological structures of heat-stressed liver tissues [84]. The histopathological changes in the hepatic tissues of the PS CON group found in this study were in line with the findings of [85]. The FO-supplemented groups showed fewer neutrophils and macrophages than those in the PS CON group. These beneficial effects could be due to FO’s antibacterial and hepatoprotective, antithrombotic, antiviral, anti-inflammatory, and antinociceptive properties [27,86].

Furthermore, the immune expression of IL1-β and TGF-β in the liver tissues was downregulated in the FO-supplemented and paracetamol groups compared to that in PS CON, while IL1-β was not expressed in the Neg. CON and FO3 groups and TGF-β was not expressed in the Neg. CON, FO2, and FO3 groups. It has been reported that essential oil supplements have modulatory effects on homeostasis, restoring the antioxidant enzyme activity to baseline levels and possibly mitigating the effects of oxidative stress [87]. The observed reduction in oxidative stress in response to dietary antioxidant supplementation is supported by scientific confirmation suggesting its ability to mitigate oxidative stress, inhibit lipid peroxidation, and reduce MDA levels [88]. Zhang et al. [89] reported that trans-anethole, the main compound in fennel oil, has anti-inflammatory and antibacterial effects. Anwar et al. [90] recorded that anethole displayed antioxidant, antibacterial, and antifungal activities. Fennel oil can act as an antioxidant by inhibiting lipid peroxidation [91]. Korver [92] reported that trans-anethole may decrease inflammatory responses and consequently their growth-inhibiting effects. Furthermore, the estragole content of FO has been highlighted for its antioxidant and anti-inflammatory activity [93]. Yu, et al. [94] explained that trans-anethole suppressed the expression of pro-inflammatory cytokines, including IL-8, IL-1β, TNF-α, and IFN-γ, but augmented the IL-10 expression in the jejunum. Adding a mixture of essential oils from citrus peels, oregano, and anise (40 mg/kg) to piglets’ diet exerted anti-inflammatory effects by lowering the expression of the NF-κB and TNF-α genes [95].

One limitation of the current study is that the acute heat stress experiment focused on a few hours and days, which may not have captured the cumulative effect of repeated heat waves or chronic stress. Future studies are recommended to evaluate the impact of graded levels of FO in alleviating chronic heat stress in broiler chickens compared to conventional treatments.

## 5. Conclusions

We concluded that fennel oil supplementation (3 g/kg diet) can mitigate the adverse effects of acute heat stress on broiler chickens’ growth performance, antioxidant, and inflammatory responses. The supplementation with FO increased the broiler chickens’ growth compared to that in the PS CON and paracetamol groups during hot temperatures. Fennel oil supplementation, especially 3 or 2 g of FO/kg diet, improved the antioxidant status of the broiler chickens, as indicated by increased CAT and SOD activity and reduced serum MDA concentrations. In addition to decreased IL1β and IFN-α, increased HSP70 concentrations, particularly in the 3 g FO/kg TRT, were observed compared to those in PS CON.

## Figures and Tables

**Table 1:** Gas chromatography–mass spectrometry (GC–MS) analysis of fennel oil.

Bioactive Compounds	Retention Time	Peak Area %
Anethole	22.86	17.44
Estragole	20.16	16.57
D-Limonene	11.72	13.41
9-Octadecenoic acid (Z)-, methyl ester	51.83	8.36
Hexadecenoic acid, methyl ester	46.56	5.19
(-)-Carvone	20.83	3.90
9,12-Octadecadienoic acid (Z,Z)-, methyl ester	51.50	3.71
Octadecanoic acid, methyl ester	52.69	1.92
Fenchone	13.32	1.06

**Table 2:** The proximate chemical composition of the diets as fed basis (%).

Ingredients (%)	Control Diet ^1^			FO 1 g/kg Diet			FO 2 g/kg Diet			FO 3 g/kg Diet		
	Starter	Grower	Finisher	Starter	Grower	Finisher	Starter	Grower	Finisher	Starter	Grower	Finisher
Yellow corn	55.725	59.25	62.2	55.725	59.25	62.2	55.725	59.25	62.2	55.725	59.25	62.2
Soybean meal, 48%	33.53	28	23.6	33.53	28	23.6	33.53	28	23.6	33.53	28	23.6
Corn gluten, 60%	4	5.325	6	4	5.325	6	4	5.325	6	4	5.325	6
Fennel oil	0	0	0	0.1	0.1	0.1	0.2	0.2	0.2	0.3	0.3	0.3
Soybean oil	2.2	3.1	4.095	2.1	3	3.995	2	2.9	3.895	1.9	2.8	3.795
Calcium carbonate	1.2	1.2	1.1	1.2	1.2	1.1	1.2	1.2	1.1	1.2	1.2	1.1
Dicalcium phosphate 18%	1.5	1.4	1.3	1.5	1.4	1.3	1.5	1.4	1.3	1.5	1.4	1.3
Nacl	0.15	0.15	0.15	0.15	0.15	0.15	0.15	0.15	0.15	0.15	0.15	0.15
Premix ^2^	0.3	0.3	0.3	0.3	0.3	0.3	0.3	0.3	0.3	0.3	0.3	0.3
DL-Methionine, 98%	0.4	0.3	0.33	0.4	0.3	0.33	0.4	0.3	0.33	0.4	0.3	0.33
Lysine HCl, 78%	0.47	0.45	0.4	0.47	0.45	0.4	0.47	0.45	0.4	0.47	0.45	0.4
Choline	0.07	0.07	0.07	0.07	0.07	0.07	0.07	0.07	0.07	0.07	0.07	0.07
L-Threonine 98.5%	0.1	0.1	0.1	0.1	0.1	0.1	0.1	0.1	0.1	0.1	0.1	0.1
Phytase	0.005	0.005	0.005	0.005	0.005	0.005	0.005	0.005	0.005	0.005	0.005	0.005
Sodium bicarbonate	0.25	0.25	0.25	0.25	0.25	0.25	0.25	0.25	0.25	0.25	0.25	0.25
Antimycotoxin	0.1	0.1	0.1	0.1	0.1	0.1	0.1	0.1	0.1	0.1	0.1	0.1
Chemical composition (%)												
ME (kcal/kg)	3003	3107	3208	3003	3107	3208	3003	3107	3208	3003	3107	3208
CP	23.12	21.50	20.02	23.12	21.50	20.02	23.12	21.50	20.02	23.12	21.50	20.02
Crude fat	4.96	5.89	6.91	4.96	5.89	6.91	4.96	5.89	6.91	4.96	5.89	6.91
Crude starch	40.62	42.97	44.90	40.62	42.97	44.90	40.62	42.97	44.90	40.62	42.97	44.90
Lysine	1.47	1.31	1.16	1.47	1.31	1.16	1.47	1.31	1.16	1.47	1.31	1.16
Methionine	0.72	0.61	0.63	0.72	0.61	0.63	0.72	0.61	0.63	0.72	0.61	0.63
Calcium	0.94	0.90	0.83	0.94	0.90	0.83	0.94	0.90	0.83	0.94	0.90	0.83
Av. P	0.48	0.45	0.42	0.48	0.45	0.42	0.48	0.45	0.42	0.48	0.45	0.42

^1^ The control diet was fed to the negative and positive control groups. ^2^ Premix per kg of diet: vitamin D3, 200 IU; vitamin A, 1 500 IU; vitamin K3, 0.5 mg; vitamin E, 10 mg; thiamine, 1.8 mg; riboflavin, 3.6 mg; folic acid, 0.55 mg; pantothenic acid, 10 mg; niacin, 35 mg; pyridoxine, 3.5 mg; biotin, 0.15 mg; cobalamin, 0.01 mg; Zn, 40 mg; Fe, 80 mg; Mn, 60 mg; Cu, 8 mg; Se, 0.15 mg I, 0.35 mg. ME: metabolizable energy; CP: crude protein; Av. P: available phosphorus.

**Table 3:** The growth performance of broiler chickens fed the experimental diets.

Items	Neg. CON	PS CON	FO1	FO2	FO3	Paracetamol	SEM	*p*-Value
Initial BW (g)	105	103	102	103	103	103	0.460	0.436
Starter period								
BW(g)	297	291	285	288	294	289	3.62	0.332
BWG (g)	192	187	183	184	191	186	3.60	0.570
FI (g)	312	307	305	307	307	306	2.73	0.082
FCR	1.63	1.65	1.67	1.66	1.61	1.65	0.013	0.881
Grower period								
BW(g)	1266 ^a^	1207 ^b^	1224 ^ab^	1204 ^b^	1184 ^b^	1204 ^b^	25.87	<0.01
BWG (g)	969 ^a^	916 ^ab^	939 ^ab^	916 ^ab^	891 ^b^	915 ^b^	25.96	<0.01
FI (g)	1275 ^ab^	1275 ^ab^	1255 ^c^	1260 ^bc^	1253 ^c^	1288 ^a^	24.45	<0.01
FCR	1.32 ^b^	1.39 ^ab^	1.34 ^ab^	1.38 ^ab^	1.41 ^a^	1.41 ^a^	0.0084	<0.01
Finisher period								
BW(g)	2421 ^a^	2147 ^c^	2215 ^b^	2205 ^b^	2188 ^b^	2176 ^bc^	32.9	<0.01
BWG (g)	1155 ^a^	940 ^c^	991 ^b^	1002 ^b^	1003 ^b^	972 ^bc^	30.0	<0.01
FI (g)	1955 ^a^	1837 ^ab^	1766 ^b^	1786 ^b^	1763 ^b^	1811 ^b^	35.4	<0.01
FCR	1.69 ^c^	1.95 ^a^	1.78 ^bc^	1.78 ^bc^	1.76 ^bc^	1.86 ^ab^	0.0176	<0.01
Overall performance								
BW(g)	2421 ^a^	2147 ^c^	2215 ^b^	2205 ^b^	2188 ^b^	2176 ^bc^	32.9	<0.01
BWG (g)	2316 ^a^	2044 ^d^	2113 ^b^	2102 ^bc^	2085 ^bc^	2073 ^cd^	31.8	<0.01
FI (g)	3542 ^a^	3418 ^ab^	3326 ^b^	3353 ^b^	3323 ^b^	3404 ^ab^	16.4	<0.01
FCR	1.53 ^c^	1.67 ^a^	1.57 ^bc^	1.60 ^bc^	1.59 ^bc^	1.64 ^ab^	0.0091	<0.01

Variation in the data was expressed as the pooled SEM. ^a, b, c^ Means within the same row carrying different superscripts significantly differ at *p* < 0.05. IBW: initial body weight; BW: body weight; BWG: body weight gain; FI: feed intake; FCR: feed conversion ratio.

**Table 4:** The effect of fennel oil and paracetamol on the meat quality of broiler chicken.

Items	Neg. CON	PS CON	FO1	FO2	FO3	Paracetamol	SEM	*p*-Value
PH	5.84	5.88	5.84	5.96	5.9	5.86	0.029	0.739
Cooking loss	25.2 ^bc^	27 ^a^	25.4 ^bc^	24.9 ^c^	25 ^c^	26.1 ^ab^	0.138	<0.01
Thawing loss	7.40 ^a^	7.06 ^b^	7.48 ^a^	7.50 ^a^	7.52 ^a^	7.48 ^a^	0.032	<0.01
Lightness	52.1 ^b^	50.4 ^c^	53.1 ^ab^	52.2 ^b^	54 ^a^	54.3 ^a^	0.347	<0.01
Redness	1.86	2.06	2.05	2.01	2.02	2.04	0.030	0.432
Yellowness	1.61 ^a^	1.52 ^b^	1.52 ^b^	1.61 ^a^	1.55 ^b^	1.51 ^b^	0.011	<0.01

Variation in the data was expressed as the pooled SEM. ^a, b, c^ Means within the same row carrying different superscripts significantly differ at *p* < 0.05.

**Table 5:** Chemical composition of breast muscles.

Items	Neg. CON	PS CON	FO1	FO2	FO3	Paracetamol	SEM	*p*-Value
Moisture %	70.5 ^d^	71.6 ^bc^	71.8 ^bc^	71.3 ^cd^	72.5 ^ab^	73.3 ^a^	0.275	<0.01
Crude protein %	19.5 ^b^	20.5 ^b^	22.8 ^a^	19.7 ^b^	20.4 ^b^	20.4 ^b^	0.341	<0.01
Fat %	3.18	2.83	2.96	2.99	2.76	3.25	0.081	0.555
Ash %	0.665 ^b^	0.980 ^a^	1.04 ^a^	0.795 ^b^	1.00 ^a^	1.09 ^a^	0.046	<0.01

Variation in the data was expressed as the pooled SEM. ^a, b, c, d^ Means within the same row carrying different superscripts significantly differ at *p* < 0.05.

**Table 6:** The biochemical indices in the blood of the broiler chickens fed the experimental diets.

Items	Neg. CON	PS CON	FO1	FO2	FO3	Paracetamol	SEM	*p*-Value
GH (ng/mL)	2.17 ^c^	2.47 ^c^	3.67 ^b^	4.4 ^ab^	4.73 ^a^	3.63 ^b^	0.234	<0.01
Glucose (mg/dL)	336	336	335	338	336	336	0.613	0.786
TP (g/dL)	3.10 ^d^	3.07 ^d^	4.16 ^bc^	4.73 ^ab^	4.99 ^a^	3.47 ^cd^	0.192	<0.01
ALB (g/dL)	1.19 ^c^	1.27 ^bc^	1.37 ^ab^	1.47 ^a^	1.49 ^a^	1.34 ^abc^	0.028	<0.01
Globulin (g/dL)	1.91 ^c^	1.79 ^c^	2.79 ^ab^	3.26 ^a^	3.50 ^a^	2.13 ^bc^	0.168	<0.01
A/G ratio	0.630 ^ab^	0.710 ^a^	0.490 ^ab^	0.450 ^b^	0.430 ^b^	0.656 ^ab^	0.031	<0.01
AST (U/L)	49.0	52.0	54.3	54.7	59.7	54.0	1.88	0.554
ALT (U/L)	6.76	7.13	7.17	8.00	8.17	8.15	0.322	0.525
Creatinine (mg/dL)	0.246	0.250	0.230	0.263	0.243	0.260	0.009	0.948
Uric acid (mg/dL)	2.96	3.00	3.02	3.23	2.99	3.03	0.069	0.924

The variation in the data was expressed as the pooled SEM. ^a, b, c, d^ Means within the same row carrying different superscripts significantly differ at *p* < 0.05. GH: growth hormone; TP: total protein; ALB: albumin.

**Table 7:** The antioxidant and inflammatory indices in broiler chickens fed the experimental diets.

Items	Neg. CON	PS CON	FO1	FO2	FO3	Paracetamol	SEM	*p*-Value
Antioxidant indices								
TAC (U/mL)	10.1 ^c^	10.1 ^c^	11.1 ^b^	11.3 ^b^	11.8 ^a^	11.1 ^b^	0.155	<0.01
CAT (U/mL)	2.13 ^c^	2.41 ^bc^	3.36 ^ab^	3.69 ^a^	4.28 ^a^	3.60 ^a^	0.194	<0.01
SOD (U/mL)	131 ^c^	131 ^c^	133 ^bc^	135 ^ab^	136 ^a^	135 ^ab^	0.569	<0.01
MDA (nmol/mL)	5.26 ^a^	4.87 ^a^	2.26 ^b^	2.94 ^b^	2.99 ^b^	2.97 ^b^	0.279	<0.01
Inflammatory indices								
IL-1β (ug/mL)	134 ^b^	151 ^a^	142 ^b^	141 ^b^	135 ^b^	139 ^b^	1.51	<0.01
IFN-α (pg/mL)	5.73 ^d^	12.3 ^a^	9.83 ^c^	11.03 ^b^	5.75 ^d^	10.7 ^bc^	0.627	<0.01
HSP70 (ng/mg)	1.70 ^b^	1.80 ^b^	2.43 ^ab^	3.13 ^b^	4.07 ^a^	3.00 ^b^	0.213	<0.01

Variation in the data was expressed as the pooled SEM. ^a, b, c, d^ Means within the same row carrying different superscripts significantly differ at *p* < 0.05. TAC: total antioxidant capacity; CAT: catalase; SOD: superoxide dismutase; MDA: malondialdehyde; IL-1β: interleukin-1-beta; IFN-α: interferon-alpha; HSP70: heat shock protein 70.

## Data Availability

The data is contained in the manuscript.

## References

[1] Diarra S.S., Tabuaciri P. (2014). Feeding management of poultry in high environmental temperatures. Int. J. Poult. Sci..

[2] Akbarian A., Michiels J., Degroote J., Majdeddin M., Golian A., De Smet S. (2016). Association between heat stress and oxidative stress in poultry; mitochondrial dysfunction and dietary interventions with phytochemicals. J. Anim. Sci. Biotechnol..

[3] Lara L.J., Rostagno M.H. (2013). Impact of heat stress on poultry production. Animals.

[4] Li M., Wu J., Chen Z. (2015). Effects of heat stress on the daily behavior of wenchang chickens. Rev. Bras. Ciência Avícola.

[5] Büyükkılıç Beyzi S., Konca Y., Kaliber M., Sarıözkan S., Kocaoğlu Güçlü B., Aktuğ E., Şentürk M. (2020). Effects of thyme essential oil and A, C, and E vitamin combinations to diets on performance, egg quality, MDA, and 8-OHdG of laying hens under heat stress. J. Appl. Anim. Res..

[6] Collier R., Renquist B., Xiao Y. (2017). A 100-Year Review: Stress physiology including heat stress. J. Dairy. Sci..

[7] Tekce E., Gül M. (2017). Effects of origanum syriacum essential oil on blood parameters of broilers reared at high ambient heat. Rev. Bras. Ciência Avícola.

[8] Tekce E., Bayraktar B., Aksakal V., Dertli E., Kamiloğlu A., Çinar Topcu K., Takma Ç., Gül M., Kaya H. (2020). Response of Japanese quails (Coturnix coturnix japonica) to dietary inclusion of Moringa oleifera essential oil under heat stress condition. Ital. J. Anim. Sci..

[9] Yilmaz E., Gul M. (2023). Effects of dietary supplementation of cumin (*Cuminum cyminum* L.) essential oil on expression of genes related to antioxidant, apoptosis, detoxification, and heat shock mechanism in heat-stressed broiler chickens. Anim. Biotechnol..

[10] Gerken M., Afnan R., Dörl J. (2006). Adaptive behaviour in chickens in relation to thermoregulation. Eur. Poult. Sci..

[11] Shahidi F., Zhong Y. (2015). Measurement of antioxidant activity. J. Funct. Foods.

[12] Abd El H.A.H.M. (2012). Lipid peroxidation end-products as a key of oxidative stress: Effect of antioxidant on their production and transfer of free radicals. Lipid Peroxidation.

[13] Nazarizadeh A., Asri-Rezaie S. (2016). Comparative study of antidiabetic activity and oxidative stress induced by zinc oxide nanoparticles and zinc sulfate in diabetic rats. AAPS PharmSciTech.

[14] Gouda A., Amer S.A., Gabr S., Tolba S.A. (2020). Effect of dietary supplemental ascorbic acid and folic acid on the growth performance, redox status, and immune status of broiler chickens under heat stress. Trop. Anim. Health Prod..

[15] Jimoh O.A., Daramola O.T., Okin-Aminu H.O., Ojo O.A. (2022). Performance, hemato-biochemical indices and oxidative stress markers of broiler chicken fed phytogenic during heat stress condition. J. Anim. Sci. Technol..

[16] Yilmaz E., Gul M. (2023). Effects of cumin (*Cuminum cyminum* L.) essential oil and chronic heat stress on growth performance, carcass characteristics, serum biochemistry, antioxidant enzyme activity, and intestinal microbiology in broiler chickens. Vet. Res. Commun..

[17] Vandana G., Sejian V., Lees A., Pragna P., Silpa M., Maloney S.K. (2021). Heat stress and poultry production: Impact and amelioration. Int. J. Biometeorol..

[18] Yilmaz E., Gul M. (2024). Effects of essential oils on heat-stressed poultry: A review. J. Anim. Physiol. Anim. Nutr..

[19] Sarıözkan S., Güçlü B.K., Konca Y., Aktuğ E., Kaliber M., Beyzi S.B., Şentürk M. (2020). The effects of thyme essential oil and vitamin combinations on performance, carcass quality and oxidation parameters in broilers exposed to heat stress. Ank. Üniversitesi Vet. Fakültesi Derg..

[20] Behairy A., Amer S.A., Gouda A., Moustafa A.A., Abdel-Warith A.-W.A., Younis E.M., Kamal A.S., Eltanahy A., Davies S.J., EL-Sayed Kamel A. (2023). Assessment of Lavandula angustifolia L. essential oil as a natural feed additive on broiler chicken’s growth, blood physiological markers, immunological status, intestinal histomorphology, and immunoexpression of CD3 and CD20. Ital. J. Anim. Sci..

[21] Amer S.A., Behairy A., Abd El-Rahman G.I., Gouda A., Abdel-Warith A.-W.A., Younis E.M., Moustafa A.A., Abdel Moniem H., Davies S.J., EL-Sayed Kamel A. (2023). Evaluation of dietary supplementation of frankincense oil on broiler chicken growth performance, hepatic histomorphology, antioxidant activity, blood biochemical parameters, and inflammatory responses. Ital. J. Anim. Sci..

[22] Amer S.A., Gouda A., Saleh G.K., Nassar A.H., Abdel-Warith A.-W.A., Younis E.M., Altohamy D.E., Kilany M.S., Davies S.J., Omar A.E. (2023). Dietary frankincense (*Boswellia serrata*) oil modulates the growth, intestinal morphology, the fatty acid composition of breast muscle, immune status, and immunoexpression of CD3 and CD20 in broiler chickens. Animals.

[23] Amer S.A., Abdel-Wareth A.A., Gouda A., Saleh G.K., Nassar A.H., Sherief W.R., Albogami S., Shalaby S.I., Abdelazim A.M., Abomughaid M.M. (2022). Impact of Dietary Lavender Essential Oil on the Growth and Fatty Acid Profile of Breast Muscles, Antioxidant Activity, and Inflammatory Responses in Broiler Chickens. Antioxidants.

[24] Abd El-Aziz A., Elfadadny A., Abo Ghanima M., Cavallini D., Fusaro I., Giammarco M., Buonaiuto G., El-Sabrout K. (2024). Nutritional value of oregano-based products and its effect on rabbit performance and health. Animals.

[25] Anka Z., Gimba S., Nanda A., Salisu L. (2020). Phytochemistry and pharmacological activities of Foeniculum vulgare. IOSR J. Pharm..

[26] Ghasemian A., Al-Marzoqi A.-H., Mostafavi S.K.S., Alghanimi Y.K., Teimouri M. (2020). Chemical composition and antimicrobial and cytotoxic activities of Foeniculum vulgare Mill essential oils. J. Gastrointest. Cancer.

[27] El-Deek A., Attia Y., Hannfy M.M. (2003). Effect of anise (*Pimpinella anisum*), ginger (*Zingiber officinale Roscoe*) and fennel (*Foeniculum vulgare*) and their mixture on performance of broilers. Eur. Poult. Sci..

[28] Hammouda F.M., Saleh M.A., Abdel-Azim N.S., Shams K.A., Ismail S.I., Shahat A.A., Saleh I.A. (2014). Evaluation of the essential oil of *Foeniculum vulgare* Mill (fennel) fruits extracted by three different extraction methods by GC/MS. Afr. J. Tradit. Complement. Altern. Med..

[29] Badgujar S.B., Patel V.V., Bandivdekar A.H. (2014). Foeniculum vulgare Mill: A review of its botany, phytochemistry, pharmacology, contemporary application, and toxicology. BioMed Res. Int..

[30] Gharaghani H., Shariatmadari F., Torshizi M. (2015). Effect of fennel (*Foeniculum vulgare* Mill.) used as a feed additive on the egg quality of laying hens under heat stress. Braz. J. Poult. Sci..

[31] Van Wyk B.-E., Wink M. (2018). Medicinal Plants of the World.

[32] Imbabi T., Sabeq I., Osman A., Mahmoud K., Amer S.A., Hassan A.M., Kostomakhin N., Habashy W., Easa A.A. (2021). Impact of fennel essential oil as an antibiotic alternative in rabbit diet on antioxidant enzymes levels, growth performance, and meat quality. Antioxidants.

[33] Roby M.H.H., Sarhan M.A., Selim K.A.-H., Khalel K.I. (2013). Antioxidant and antimicrobial activities of essential oil and extracts of fennel (*Foeniculum vulgare* L.) and chamomile (*Matricaria chamomilla* L.). Ind. Crops Prod..

[34] Rather M.A., Dar B.A., Sofi S.N., Bhat B.A., Qurishi M.A. (2016). Foeniculum vulgare: A comprehensive review of its traditional use, phytochemistry, pharmacology, and safety. Arab. J. Chem..

[35] AVIAGEN Ross Broiler Nutrition Specification 2014. https://aviagen.com/.

[36] AVMA (2013). AVMA Guidelines for the Euthanasia of Animals: 2013 Edition.

[37] Petracci M., Baéza E. (2011). Harmonization of methodologies for the assessment of poultry meat quality features. World’s Poult. Sci. J..

[38] AOAC (2000). Official Methods of Analysis of AOAC International.

[39] Trinder P. (1969). Determination of blood glucose using an oxidase-peroxidase system with a non-carcinogenic chromogen. J. Clin. Pathol..

[40] Henry R. (1974). Determination of Serum Creatinine. Clinical Chemistry: Principles and Techniques.

[41] Sanders G., Pasman A., Hoek F. (1980). Determination of uric acid with uricase and peroxidase. Clin. Chim. Acta.

[42] Reitman S., Frankel S. (1957). Determination of serum glutamic oxaloacetic transaminase and pyruvic transaminase by colorimetric method. Am. J. Clin. Pathol..

[43] Grant G. (1987). Amino acids and proteins. Fundamentals of Clinical Chemistry.

[44] Doumas B., Baysa D., Carter R., Peters T., Schaffer R. (1981). Determination of serum total protein. Clin. Chem..

[45] Doumas B., Biggs H., Cooper G.R. (1972). Determination of Serum Albumin in Standard Method of Clinical Chemistry.

[46] Rice-Evans C., Miller N.J. (1994). [241 Total antioxidant status in plasma and body fluids. Methods Enzymol..

[47] Aebi H. (1984). Catalase in vitro. Methods in Enzymology.

[48] Nishikimi M., Rao N.A., Yagi K. (1972). The occurrence of superoxide anion in the reaction of reduced phenazine methosulfate and molecular oxygen. Biochem. Biophys. Res. Commun..

[49] Mcdonald R.E., Hultin H.O. (1987). Some characteristics of the enzymic lipid peroxidation system in the microsomal fraction of flounder skeletal muscle. J. Food Sci..

[50] Anderson R., Wang C., Van Kersen I., Lee K., Welch W., Lavagnini P., Hahn G. (1993). An immunoassay for heat shock protein 73/72: Use of the assay to correlate HSW3/72 levels in mammalian cells with heat response. Int. J. Hyperth..

[51] Suvarna S., Layton C., Bancroft J.D. (2013). Theory and Practice of Histological Techniques.

[52] Saber S., Khalil R.M., Abdo W.S., Nassif D., El-Ahwany E. (2019). Olmesartan ameliorates chemically-induced ulcerative colitis in rats via modulating NFκB and Nrf-2/HO-1 signaling crosstalk. Toxicol. Appl. Pharmacol..

[53] Rizzardi A.E., Johnson A.T., Vogel R.I., Pambuccian S.E., Henriksen J., Skubitz A.P., Metzger G.J., Schmechel S.C. (2012). Quantitative comparison of immunohistochemical staining measured by digital image analysis versus pathologist visual scoring. Diagn. Pathol..

[54] Attia Y.A., Al-Harthi M.A., Sh. Elnaggar A. (2018). Productive, physiological and immunological responses of two broiler strains fed different dietary regimens and exposed to heat stress. Ital. J. Anim. Sci..

[55] Attia Y.A., Hassan S.S. (2017). Broiler tolerance to heat stress at various dietary protein/energy levels. Eur. Poult. Sci./Arch. Für Geflügelkunde.

[56] Butcher G., Miles R.D. (2002). Interrelationship of Nutrition and Immunity.

[57] Bayraktar B., Tekce E., Aksakal V., Gül M., Takma Ç., Bayraktar S., Bayraktar F.G., Eser G. (2020). Effect of the addition of essential fatty acid mixture to the drinking water of the heat stress broilers on adipokine (Apelin, BDNF) response, histopathologic findings in liver and intestines, and some blood parameters. Ital. J. Anim. Sci..

[58] Madkour M., Salman F.M., El-Wardany I., Abdel-Fattah S.A., Alagawany M., Hashem N.M., Abdelnour S.A., El-Kholy M.S., Dhama K. (2022). Mitigating the detrimental effects of heat stress in poultry through thermal conditioning and nutritional manipulation. J. Therm. Biol..

[59] Brenes A., Roura E. (2010). Essential oils in poultry nutrition: Main effects and modes of action. Anim. Feed. Sci. Technol..

[60] Platel K., Srinivasan K. (2001). Studies on the influence of dietary spices on food transit time in experimental rats. Nutr. Res..

[61] Al-Sagan A.A., Khalil S., Hussein E.O., Attia Y.A. (2020). Effects of fennel seed powder supplementation on growth performance, carcass characteristics, meat quality, and economic efficiency of broilers under thermoneutral and chronic heat stress conditions. Animals.

[62] Schöne F., Vetter A., Hartung H., Bergmann H., Biertümpfel A., Richter G., Müller S., Breitschuh G. (2006). Effects of essential oils from fennel (*Foeniculi aetheroleum*) and caraway (*Carvi aetheroleum*) in pigs. J. Anim. Physiol. Anim. Nutr..

[63] Mohammed A.A., Abbas R.J. (2009). The effect of using fennel seeds (*Foeniculum vulgare* L.) on productive performance of broiler chickens. Int. J. Poult. Sci..

[64] Singh P., Mishra N., Gupta E. (2020). Phytochemistry and ethanopharmacology of *Illicium verum* (*Staranise*). Ethnopharmacological Investigation of Indian Spices.

[65] Fletcher D. (2002). Poultry meat quality. World’s Poult. Sci. J..

[66] Sandercock D., Hunter R.R., Nute G.R., Mitchell M., Hocking P.M. (2001). Acute heat stress-induced alterations in blood acid-base status and skeletal muscle membrane integrity in broiler chickens at two ages: Implications for meat quality. Poult. Sci..

[67] Wang R., Liang R., Lin H., Zhu L., Zhang Y., Mao Y., Dong P., Niu L., Zhang M., Luo X. (2017). Effect of acute heat stress and slaughter processing on poultry meat quality and postmortem carbohydrate metabolism. Poult. Sci..

[68] Mir N.A., Rafiq A., Kumar F., Singh V., Shukla V. (2017). Determinants of broiler chicken meat quality and factors affecting them: A review. J. Food Sci. Technol..

[69] CIE Technical Report: Colorimetry, Commission Internationale de l’Éclairage Central Bureau. Vienna, Austria 2004. https://cielab.xyz/pdf/cie.15.2004%20colorimetry.pdf.

[70] Amorati R., Foti M.C., Valgimigli L. (2013). Antioxidant activity of essential oils. J. Agric. Food Chem..

[71] Miyashita K. (2014). Paradox of omega-3 PUFA oxidation. Eur. J. Lipid Sci. Technol..

[72] Brewer M. (2009). Irradiation effects on meat flavor: A review. Meat Sci..

[73] Smith D., Northcutt J. (2003). Red discoloration of fully cooked chicken products. J. Appl. Poult. Res..

[74] Belhadj Slimen I., Najar T., Ghram A., Abdrrabba M. (2016). Heat stress effects on livestock: Molecular, cellular and metabolic aspects, a review. J. Anim. Physiol. Anim. Nutr..

[75] Wetterling T., Veltrup C., Driessen M., John U. (1999). Drinking pattern and alcohol-related medical disorders. Alcohol. Alcohol..

[76] Kumar A. (2012). A review on hepatoprotective herbal drugs. Int. J. Res. Pharm. Chem..

[77] Nazir T., Shakir L., Rahman Z.-u., Najam K., Choudhary A., Saeed N., Rasheed H., Nazir A., Aslam S., Khanum A. (2020). Hepatoprotective activity of Foeniculum vulgare against paracetamol induced hepatotoxicity in rabbit. J. Appl. Pharm..

[78] Ciftci M., UG S., MA A., IH C., Tonbak F. (2013). The effects of dietary rosemary (*Rosmarinus officinalis* L.) oil supplementation on performance, carcass traits and some blood parameters of Japanese quail under heat stressed condition. Kafkas Üniversitesi Vet. Fakültesi Derg..

[79] Nawaz A.H., Amoah K., Leng Q.Y., Zheng J.H., Zhang W.L., Zhang L. (2021). Poultry response to heat stress: Its physiological, metabolic, and genetic implications on meat production and quality including strategies to improve broiler production in a warming world. Front. Vet. Sci..

[80] Emami N.K., Jung U., Voy B., Dridi S. (2020). Radical response: Effects of heat stress-induced oxidative stress on lipid metabolism in the avian liver. Antioxidants.

[81] Balakrishnan K.N., Ramiah S.K., Zulkifli I. (2023). Heat shock protein response to stress in poultry: A review. Animals.

[82] Valko M., Leibfritz D., Moncol J., Cronin M.T., Mazur M., Telser J. (2007). Free radicals and antioxidants in normal physiological functions and human disease. Int. J. Biochem. Cell Biol..

[83] Tang L.-P., Liu Y.-L., Zhang J.-X., Ding K.-N., Lu M.-H., He Y.-M. (2022). Heat stress in broilers of liver injury effects of heat stress on oxidative stress and autophagy in liver of broilers. Poult. Sci..

[84] Nawaz A.H., Lin S., Wang F., Zheng J., Sun J., Zhang W., Jiao Z., Zhu Z., An L., Zhang L. (2023). Investigating the heat tolerance and production performance in local chicken breed having normal and dwarf size. Animal.

[85] Adu-Asiamah P., Zhang Y., Amoah K., Leng Q., Zheng J., Yang H., Zhang W., Zhang L. (2021). Evaluation of physiological and molecular responses to acute heat stress in two chicken breeds. Animal.

[86] Guimarães R., Barros L., Carvalho A.M., Ferreira I.C. (2011). Infusions and decoctions of mixed herbs used in folk medicine: Synergism in antioxidant potential. Phytother. Res..

[87] Elbaz A.M., Ashmawy E.S., Salama A.A., Abdel-Moneim A.-M.E., Badri F.B., Thabet H.A. (2022). Effects of garlic and lemon essential oils on performance, digestibility, plasma metabolite, and intestinal health in broilers under environmental heat stress. BMC Vet. Res..

[88] Azad M., Kikusato M., Maekawa T., Shirakawa H., Toyomizu M. (2010). Metabolic characteristics and oxidative damage to skeletal muscle in broiler chickens exposed to chronic heat stress. Comp. Biochem. Physiol. Part A Mol. Integr. Physiol..

[89] Zhang S., Chen X., Devshilt I., Yun Q., Huang C., An L., Dorjbat S., He X. (2018). Fennel main constituent, trans-anethole treatment against LPS-induced acute lung injury by regulation of Th17/Treg function. Mol. Med. Rep..

[90] Anwar F., Hussain A.I., Sherazi S.T.H., Bhanger M.I. (2009). Changes in composition and antioxidant and antimicrobial activities of essential oil of fennel (*Foeniculum vulgare* Mill.) fruit at different stages of maturity. J. Herbs Spices Med. Plants.

[91] Mohamad R.H., El-Bastawesy A.M., Abdel-Monem M.G., Noor A.M., Al-Mehdar H.A.R., Sharawy S.M., El-Merzabani M.M. (2011). Antioxidant and anticarcinogenic effects of methanolic extract and volatile oil of fennel seeds (*Foeniculum vulgare*). J. Med. Food.

[92] Korver D. (2012). Implications of changing immune function through nutrition in poultry. Anim. Feed Sci. Technol..

[93] Mahendra M.Y., Purba R.A., Dadi T.B., Pertiwi H. (2023). Estragole: A review of its pharmacology, effect on animal health and performance, toxicology, and market regulatory issues. Iraqi J. Vet. Sci..

[94] Yu C., Wang D., Tong Y., Li Q., Yang W., Wang T., Yang Z. (2022). Trans-anethole alleviates subclinical necro-haemorrhagic enteritis-induced intestinal barrier dysfunction and intestinal inflammation in broilers. Front. Microbiol..

[95] Kroismayr A., Sehm J., Pfaffl M., Schedle K., Plitzner C., Windisch W. (2008). Effects of avilamycin and essential oils on mRNA expression of apoptotic and inflammatory markers and gut morphology of piglets. Czech J. Anim. Sci..

